# Adrenal venous sampling in primary hyperaldosteronism: correlation of hormone indices and collimated C-arm CT findings

**DOI:** 10.1007/s00261-021-03003-0

**Published:** 2021-03-05

**Authors:** L. S. Becker, M. H. Hinrichs, T. Werncke, C. L. A. Dewald, S. K. Maschke, F. P. Limbourg, K. I. Ringe, J. B. Hinrichs, F. Wacker, B. C. Meyer

**Affiliations:** 1 Department of Diagnostic and Interventional Radiology, Institute for Diagnostic and Interventional Radiology, Hannover Medical School, OE8220, Carl-Neuberg-Str. 1, 30625 Hannover, Germany; 2 Department of Nephrology and Hypertension, Hannover Medical School, Hannover, Germany

**Keywords:** Adrenal venous sampling, Collimated C-arm CT, Primary hyperaldosteronism, Radiation exposure

## Abstract

**Purpose:**

To evaluate the feasibility and effect of an approach to adrenal venous sampling (AVS) analysis by combining established selective cortisol and aldosterone indices with the acquisition of a collimated C-arm CT(CACT_Coll_).

**Methods:**

Overall, 107 consecutive patients (45f,62 m; 54 ± 10 years) undergoing 111 AVS procedures without hormonal stimulation from 7/13 to 2/20 in a single institution were retrospectively analysed. Hormone levels were measured in sequential samples of the suspected adrenal veins and right iliac vein, and selectivity indices (SI) computed. Stand-alone SI_Cortisol_ and/or SI_Aldosterone_ ≥ 2.0 as well as SI_Cortisol_ and/or SI_Aldosterone_ ≥ 1.1 combined with positive right-sided CACT_Coll_ of the adrenals (*n* = 80; opacified right adrenal vein) were defined as a successful AVS procedure. Radiation exposure of CACT was measured via dose area product (DAP) and weighed against an age-/weight-matched cohort (*n* = 66).

**Results:**

Preliminary success rates (SI_Cortisol_ and/or SI_Aldosterone_ ≥ 2.0) were 99.1% (left) and 72.1% (right). These could be significantly increased to a 90.1% success rate on the right, by combining an adjusted SI of 1.1 with a positive CACT_Coll_ proving the correct sampling position. Sensitivity for stand-alone collimated CACT (CACT_Coll_) was 0.93, with 74/80 acquired CACT_Coll_ confirming selective cannulation by adrenal vein enhancement. Mean DAP_Coll_CACT_ measured 2414 ± 958 μGyxm^2^, while mean DAP_Full-FOV_CACT_ in the matched cohort measured 8766 ± 1956 μGyxm^2^ (p < 0.001).

**Conclusion:**

Collimated CACT in AVS procedures is feasible and leads to a significant increase in success rates of (right-sided) selective cannulation and may in combination with adapted hormone indices, offer a successful alternative to previously published AVS analysis algorithms with lower radiation exposure compared to a full-FOV CACT.

## Introduction

Primary hyperaldosteronism (PA) has nowadays emerged as the most common cause of endocrine hypertension with a reported prevalence of > 10% and strong evidence of an increased risk of cardiorenal complications following hypertension-induced damage to numerous organs [1–6]. The majority of patients are diagnosed with either unilateral aldosterone-producing adenoma (APA) or bilateral adrenal hyperplasia (BAH) [1–4, 6–11]. With fundamentally different therapeutic strategies and outcomes, distinguishing the subtypes and patients adequately treated by adrenalectomy becomes crucial. While BAH is typically treated with aldosterone antagonists in combination with other anti-hypertensive drugs [2, 5, 6], unilateral APA can be cured by unilateral adrenalectomy, emphasizing the significance of reliable diagnostic tools such as adrenal venous sampling (AVS) for early identification. AVS, a procedure for the selective evaluation of the plasma (or serum) aldosterone concentration (PAC) and plasma cortisol concentration (PCC) in the adrenal veins (AV), has been recommended as gold standard by the guidelines of the Endocrine Society [1, 3, 9, 12, 13], with reported sensitivity and specificity values of $\ge$ 90% in specialized and well-trained centers [1, 9, 12, 14–16]. In spite of the guideline recommendations and compelling evidence however, a strong perception of AVS as a difficult, complex, risky and occasionally unnecessary procedure with the threat of repeat testing in case of catheter malpositioning, is reported in the literature [1, 2, 4, 6–11, 13, 17]. Success of AVS has been reported to be dependent on the level of experience of the interventional radiologist and knowledge of the adrenal anatomy, especially due to the small dimensions of the right adrenal vein (RAV) and the multitude of anatomical variations, draining directly into the inferior vena cava (IVC) at differing angles [1, 15, 16]. Failure of selective cannulation may lead to repetition of the intervention, increased radiation exposure for both patient and operator, or even inaccurate diagnosis. Recent studies report a benefit from the use of quick cortisol assays (QCA) for peri-procedural cortisol assessment, especially in centers with low success rates [18–20]. However, this requires the availability of dedicated equipment as well as trained personnel. Moreover, a negative QCA offers no additional anatomic information concerning the origin of the adrenal vein and possible evaluation of a more suitable catheter position. Another branch of studies reported significantly increased success rates by confirming catheter position with ancillary imaging such as full-FOV C-arm CT (CACT_Full-FOV_) [7, 8, 12, 21, 22]. In comparison with the binary feedback derived from a QCA (correct vs. incorrect catheter position), multifaceted information may be gained from 3D imaging, especially in interventionalists with a wide variety of experience. To balance diagnostic success with limited radiation exposure to patient and operator, we adopted a size-adjusted collimation CACT in our center instead of previously published full field of view CACT [7, 8, 12, 21, 22]. The purpose of this study was to evaluate the feasibility and clinical benefit of collimated CACT in conjunction with the measured cortisol and aldosterone concentrations in AVS.

## Materials and methods

### Patient selection

This retrospective single-center study was approved by the institutional review board of our hospital. The indication for the procedure was consensually obtained with the department of Nephrology and Hypertension. From July 2013 to February 2020, 118 consecutive AVS procedures in 114 patients were performed at our hospital. Of these 118 interventions, seven were excluded due to premature discontinuation of the procedure by patients unwilling to undergo the complete procedure (*n* = 6) or accidental pre-procedural receipt of cortisone (*n* = 1), with the remaining 111 interventions in 107 patients (45 females, 62 males; 54 ± 10 years) composing our study group. Pre-interventional mean systolic blood pressure was 160 ± 24 mmHg, mean diastolic blood pressure 94 ± 14 mmHg, the mean number of blood pressure medications lay at 3.7 and 56 (49%) exhibited symptoms of low potassium, of which 41 (73%) needed to be substituted. Sampling was performed in our current angiography suites, containing a ceiling-mounted as well as a robotic-arm-mounted angiography system (Artis Q®, Artis pheno®, Siemens Healthcare GmbH, Forchheim, Germany). To ensure the correct sampling position, 80 collimated CACTs were performed. Specific patient chracteristics are summarized in Table 1.

**Table 1:** Patient’s demographics and interventional data

	AVS cohort July 2013–February 2020
Interventions	118
Included	111
Excluded	7
Premature procedural cancellation	6
Receipt of prednisolone	1
Patients	114
Included (m,f)	107 (63, 45)
Excluded (m,f)	7 (3, 4)
Gender	
Male (%)	62 (58%)
Female (%)	45 (42%)
Age	55 ± 10
BMI	29 ± 6
AVS: successful catheterization	
Right: SI_Cortisol_ ≥ 2.0	80 (72.1%)
SI_Aldosterone_ ≥ 2.0	81 (73%)
Positive CACT_Coll_ + SI_Cortisol, Aldosterone_ ≥ 1.1	100 (90.1%)
Left: SI_Cortisol/Aldosterone_ ≥ 2.0	110 (99.1%)
SI_Aldosterone_ ≥ 2.0	92 (82.9%)
Imaging	
CACT_Coll_	80 (72.1%)
Positive	74 (92.5%)
Negative	6 (7.5%)
Plasma values	
Cortisol_AV_	
Right	198 ± 289 [8–1070]
Left	136 ± 197 [2–930]
Cortisol_PV_	
Right	14 ± 10 [4–26]
Left	12 ± 6 [2.4–930]
Aldosterone_AV_	
Right	6039 ± 21,461 [61–177000]
Left	3331 ± 8292 [151–70300]
Aldosterone_PV_	
Right	240 ± 261 [30–2180]
Left	232 ± 284 [0.7–35]
SI_Cortisol_	
Right	13.5 ± 17.5 [0.7–56]
Left	10 ± 12 [0.8–72]
SI_Aldosterone_	
Right	21 ± 38 [0.8–108]
Left	18 ± 31 [0.6–158]

Continuous data are presented as means ± standard deviation (range if available); categorical data are given as the counts/sample (percentage)

*AV* adrenal vein; *AVS* adrenal vein sampling; BMI, *CACT* C-arm CT; *DSA* digital subtraction angiography; *f* female; *m* male; *PV* peripheral vein; *SI* selectivity index

### Adrenal vein sampling protocol

**Fig. 1:**
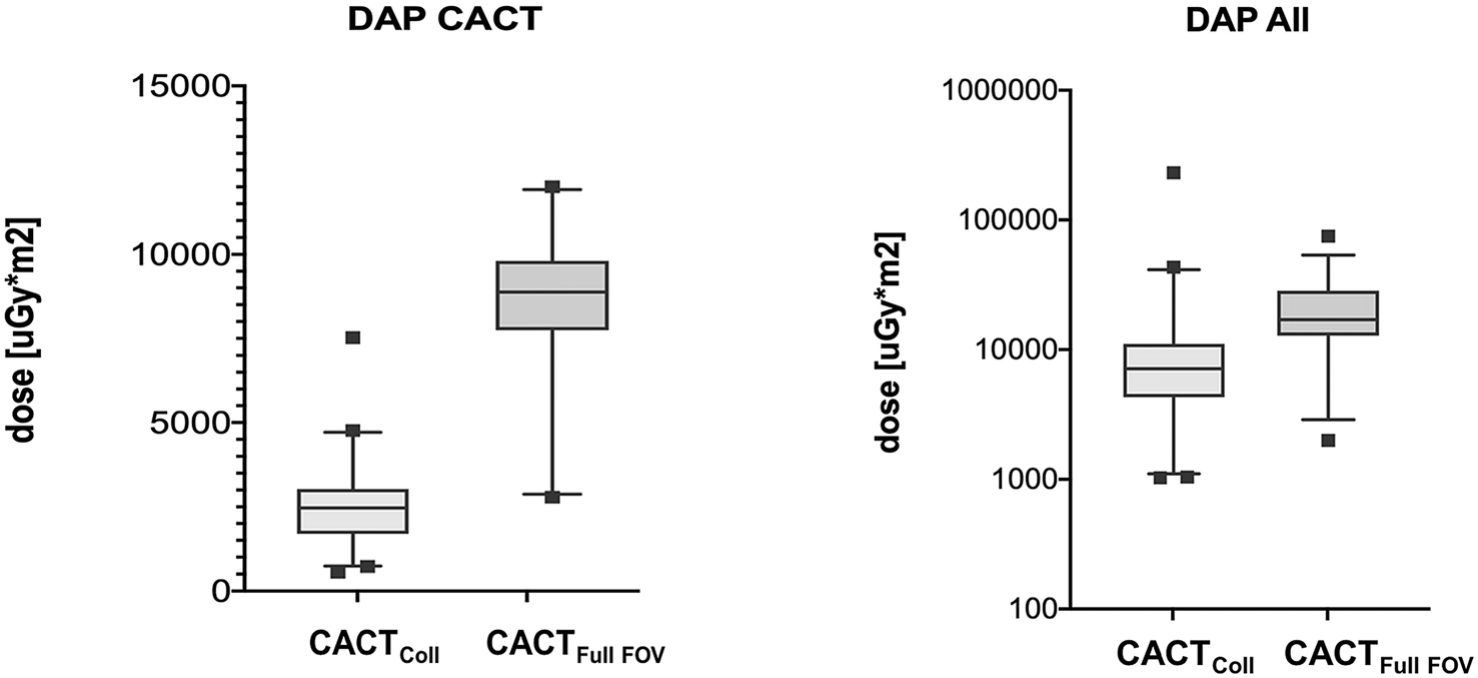
**a**, **b** Boxplot presentation of dose area products (DAP), comparing collimated vs. full-FOV CACTs in our AVS cohort with those of an age- and weight-matched cohort undergoing liver-directed therapies. DAP_All_ consists of CACT, fluoroscopy, and DSA dosage values

**Fig. 2:**
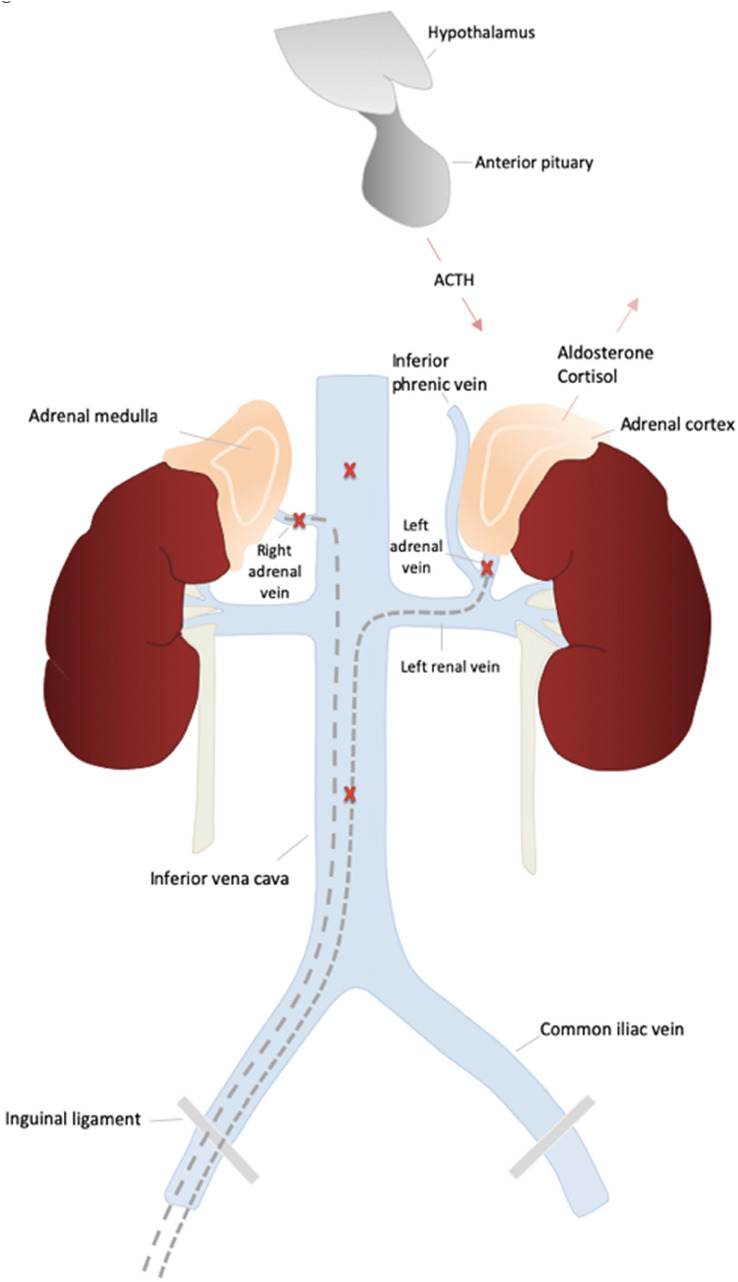
Schematic diagram of adrenal venous sampling and the interaction of the hypothalamic–pituitary–adrenal axis components. After local anesthesia, a 6F introducer sheath was inserted into the right femoral vein under sonographic guidance; appropriate angiographic catheters were then used to selectively catheterize the adrenal veins. Selective blood samples were carefully collected from the adrenal veins and from the supra- and infrarenal sections of the IVC (red x). In addition, simultaneous blood samples from the sheath were taken, serving as reference

**Fig. 3:**
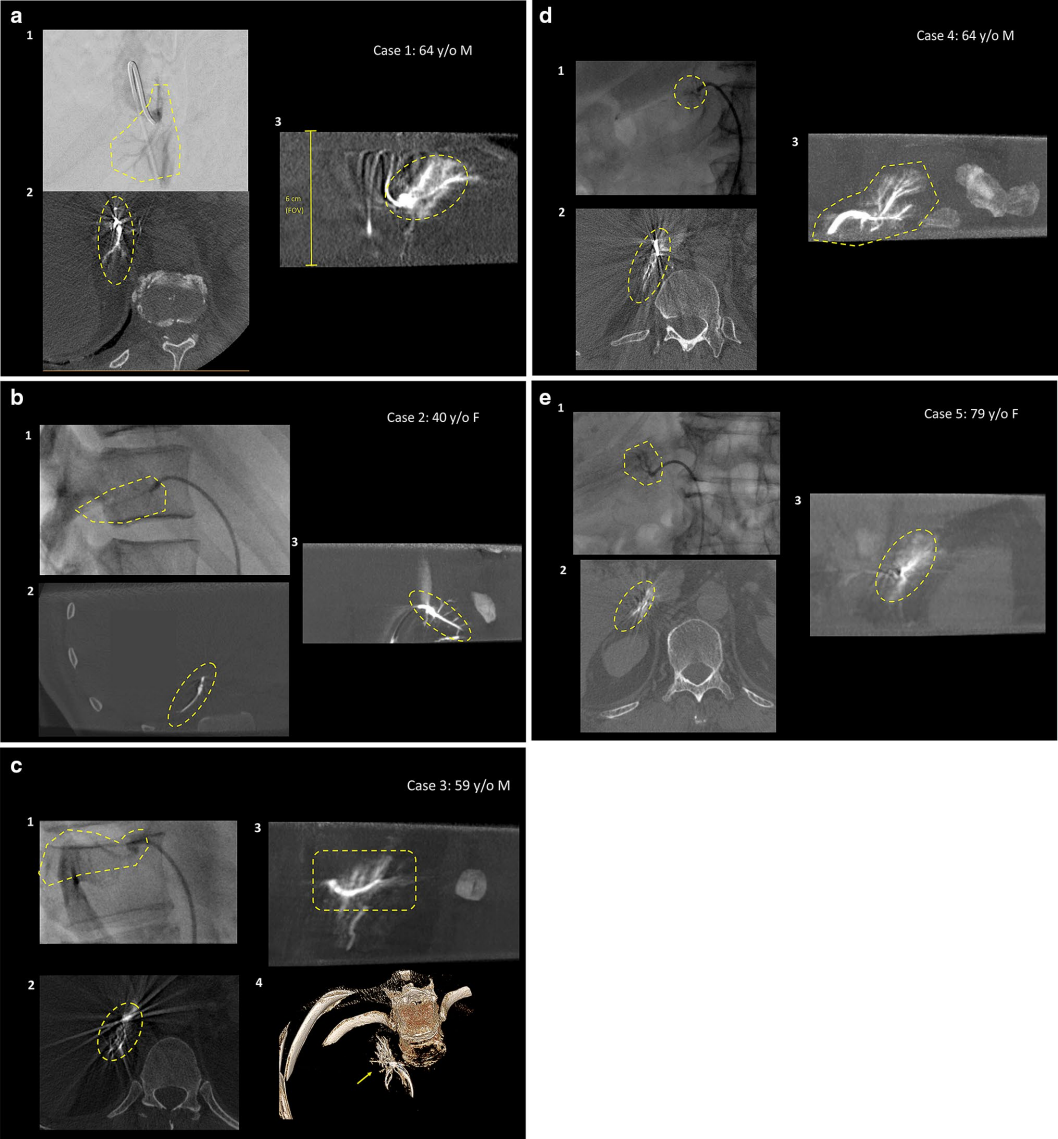
**a–e** Five cases of CACT-assisted adrenal vein sampling. Frontal view of a digital subtraction angiography (**A.1**) obtained with contrast injection via Sos Omni catheter and subsequent demonstration of a typical opacification of the adrenal veins, located anterolaterally of the vertebral body L1. Collimated CACT (**A.2**: axial view; **A.3**: sagittal maximum intensity projection of 5 mm (MIP)) proves correct catheter position by selective opacification of the right adrenal. The yellow perpendicular line **(A.3)** demonstrates the size of the collimated CACT’s field of view (6 cm). **b** Lateral view of a fluoroscopic image (**B.1**) obtained with contrast injection via 4F Cobra catheter and subsequent demonstration of a weak opacification of the supposed adrenal veins. Performance of additional collimated CACT (**B.2**: axial view; **B.3**: sagittal maximum intensity projection of 5 mm (MIP)) confirms correct catheter position by selective opacification of the right adrenal. **c** Lateral view of a fluoroscopic image (**C.1**) obtained with contrast injection via 5F Cobra catheter and subsequent demonstration of a weak opacification of the supposed adrenal veins. Performance of additional collimated CACT (**C.2**: axial view; **C.3**: sagittal maximum intensity projection of 5 mm (MIP), **C.4**. Volume Rendering Technique (VRT)) confirms correct catheter position by selective opacification of the right adrenal (yellow arrow). **d** Frontal view of a fluoroscopic image (**D.1**) obtained with contrast injection via 5F Cobra catheter and subsequent demonstration of a weak opacification of the supposed adrenal veins. Performance of additional collimated CACT (**D.2**: axial view; **D.3**: sagittal maximum intensity projection of 5 mm (MIP)) confirms correct catheter position by selective opacification of the right adrenal (yellow arrow). **e** Lateral view of a fluoroscopic image (**E.1**) obtained with contrast injection via 4F Cobra catheter and subsequent demonstration of a weak opacification of the supposed adrenal veins. Performance of additional collimated CACT (**E.2**: axial view; **E.3**: sagittal maximum intensity projection of 5 mm (MIP)) confirms correct catheter position by selective opacification of the right adrenal (yellow arrow)

AVS procedures were scheduled for early to mid-morning (7–11 a.m.) to avoid the expected lower adrenal cortisol excretion later in the day [3, 5, 15]. After local anesthesia, a 6F introducer sheath was inserted into the right femoral vein under sonographic guidance; appropriate angiographic catheters were then used to selectively catheterize the adrenal veins. AVS were taken sequentially and without concomitant adrenocorticotropic hormone (ACTH) stimulation. The catheter most often selected by the 5 performing interventional radiologists (2- > 10 years of experience), based on the patient’s individual anatomic circumstances, was a Cobra catheter, followed by SosOmni, Sidewinder 1/2, and Shepherd Hook. Assessment of catheter position was obtained fluoroscopically with gentle manual injection of contrast media, after selective blood samples were carefully collected from the adrenal veins and from the supra- and infrarenal sections of the IVC (see Fig. 1). In addition, simultaneous blood samples from the sheath were taken, serving as reference. Our standard approach is to obtain three samples from the suspected veins. In case of a distinct sampling position less samples were taken—in unclear case more samples were needed. Thus, the mean number of samples from the suspected adrenal veins lay at 3 [2;5] for the right and 2 [1;3] for the left side. In 80 cases of ambiguous catheter position on the right side, the interventional radiologists used acquisition of collimated CACTs to confirm the sampling position, defined as enhancement of the adrenal gland post-contrast. Subsequently, the in-house laboratory institute assessed the cortisol and aldosterone concentrations. After data acquisition, radiological images, laboratory results and medical records were analyzed for image findings, potential complications, and technical and biochemical success rates (Figs. 2, 3).

### Collimated C-Arm CT

All 80 CACTs in the main patient cohort were acquired during simultaneous gentle contrast injection by hand and were centered on the tip of the angiography catheter. Contrast medium (CM) was diluted 1:1 in normal saline to minimize hardening artifacts. CACTs were acquired during a modest breath-hold of the patient executing the manufacturer’s preset (Artis Q: 6 s DR DynaCT® preset, Siemens; Artis pheno®: 5 s DR DynaCT® preset, Siemens). Craniocaudal coverage of the collimated CACT lay at approximately 6 cm. After acquisition, the CACT dataset was automatically sent to a dedicated workstation (syngo X Workplace® VD10C, Siemens) and 3D multiplanar reconstruction images were obtained. Catheter position was checked and adapted if necessary.

### AVS-derived indices and definition of successful catheterization

The selectivity index (SI) is defined as the ratio of the measured plasma cortisol concentration in the respective adrenal vein to that of the peripheral vein (SI_Cortisol_ = PCC_AV_ / PCC_PV_). In addition, an equivalent index was computed for aldosterone (SI_Aldosterone_ = PAC_AV_ / PAC_PV_), to give consideration to situations of lateralizing adenoma, in which contralateral aldosterone secretion was not completely suppressed according to Mailhot et al. [23]. Primary successful catheterization of the adrenal vein was defined by a SI_Cortisol_ ≥ 2.0 or a SI_Aldosterone_ ≥ 2.0. In case of confirmed adrenal catheter position via CACT_Coll_, the SI_Cortisol_ or SI_Aldosterone_ was adapted to ≥ 1.1, in order to prevent false dismissal of values lower than the initial cutoff, attributable to suppression. An internal validation of the centrally measured hormone concentration, independent of the circadian fluctuations, could be achieved by interpreting differences of more than two-fold standard deviations as pathologic. For the purpose of potential sub-classification, computation of the lateralization index (LI) may be performed, defined as the ratio of the aldosterone/cortisol concentration on the dominant side to that on the contralateral side ((PAC_AV dominant_ / PCC_AV dominant_): (PAC_AV nondominant_ / PCC_AV nondominant_)). While a LI ≥ 2.0 (as used in this study) has been reported to describe unilateral disease, leading to further categorization into right-dominant, left-dominant, or non-lateral, a LI > 3 indicates the probability of APA [1, 5, 21]. Upon failure to selectively cannulate one side, computation of the contralateral suppression index (CSI) may sometimes indirectly indicate the presence of an APA on the contralateral side, as it may indicate unilateral disease if adrenal hormone values present lower than in the periphery [1, 5]. The CSI is calculated by dividing the aldosterone/cortisol ratio on the nondominant side by that in the peripheral vein ((PAC_AV nondominant_ / PCC_AV nondominant_): (PAC_PV_ / PCC_PV_)). All aforementioned ratios are summarized in Table 2.

**Table 2:** Definition and clinical significance of selectivity index, Lateralization index, and contralateral suppression index, cutoff values

Definition	Formula	Cutoff values (no ACTH stimulation)		Clinical significance
		*w/o CACT*	*w/pos.CACT*	
Selectivity index				Blood sample was obtained correctly from AV, if the computed value is greater than the cutoff
Cortisol	PCC_AV_/PCC_PV_	≥ 2.0	≥ 1.1	
Aldosterone	PAC_AV_/PAC_PV_	≥ 2.0	≥ 1.1	
Lateralization index	(PAC/ PCC)_AV dom_: (PAC/ PCC)_AV nondom_	≥ 2.0		Lateralized aldosterone excess is demonstrated by values greater than the cutoff
Contralateral suppression index	(PAC/PCC)_AV nondom_: (PAC/PCC)_PV_			Ipsilateral suppression in combination with contralateral aldosterone overproduction may be indicated by adrenal values lower than in the periphery

*AV* Adrenal vein; *dom* dominant side; *nondom* nondominant side; *PAC* plasma aldosterone concentration; *PAC* Plasma aldosterone concentration; *PCC* plasma cortisol concentration; *pos.* positive; *PV* peripheral vein

### Radiation exposure analysis

To evaluate radiation exposure, the dose–area product (DAP_All_) was documented for each patient, consisting of DAP for CACT, fluoroscopy, and DSA in standard AVS procedures. The fluoroscopy time was also recorded. To assess the potential reduction of radiation exposure by CACT collimation, a comparable, age- and weight-matched cohort of 66 patients undergoing liver-directed therapies was compiled, receiving a full-FOV CACT on the same angiography systems at our institution. Means for DAP_CACT_ as well as DAP_Full-FOV_CACT_ and fluoroscopy time were computed and compared (see Table 3).

**Table 3:** Comparison of age, BMI, dose values, and fluoroscopy time [mean ± SD] in collimated CACT (main cohort) and full-FOV CACT (control group) via table and boxplot presentation

	AVS cohort (CACT_Coll_)	Control group (CACT_Full FOV_)	p-value
Number of interventions	80	66	
Age	55 ± 10	65 ± 10	
BMI	29 ± 6	28 ± 5	0.053
DAP_All_	8397 ± 6507	21,522 ± 12,617	
DAP_CACT_	2486.6 ± 1129.2	8766.1 ± 1956	< 0.001
Fluoroscopy time [hh:mm:ss]	00:23:03	00:23:00	

### Statistical analysis

Descriptive statistical analyses of interventional data and the patient demographics were computed and are presented as means ± standard deviations (SD). Comparisons between data were made using an unpaired t-test as well as paired Wilcoxon signed-rank test as appropriate. All statistical analyses were performed using commercially available software (IBM SPSS Statistics, version 26, 64-bit version). A *p*-value < 0.05 was considered significant.

## Results

After separate analysis of both sides, 110 of the included 111 interventions attained a SI_Cortisol_ > 2.0 on the left and 80 on the right, corresponding to preliminary success rates of 99.1% and 72.1%, respectively. Sensitivity of singular SI_Aldosterone_ > 2.0 lay at 0.83 (left) and 0.73 (right). By expanding this single criterion to a combination of factors, i.e. proven imaging success via collimated CACT and adaptation of hormone indices to ≥ 1.1, adrenal vein cannulation of the right AV could be confirmed in 20 additional patients, totaling 100 cases of selective catheterization on the right (90.1%). The majority of the seven total indices that did not reach the formal cutoff values, pertained to the right AV (*n* = 6), while 1 pertained to the left. Of these, three did not receive a CACT, and with samples proving inconclusive, were interpreted as catheter malposition at time of sample, while the remaining four showed catheter malposition in accessory hepatic veins in the CACTs. Correlation with the 38 patients having received surgery at our center amounted to 38 cases, i.e. 32 adenomas, two microadenomas and four cases of adrenal cortex hyperplasia were diagnosed. Of these, the majority demonstrated SI_Cortisol_ ± SI_Aldosterone_ > 2.0; five cases with histologically proven adrenal pathology with SI_Cortisol, Aldosterone_ ≥ 1.1 had a positive CACT. In none of the patients with available pathology results, a SI_Cortisol, Aldosterone_ ≤ 2.0 and receipt of a CACT, CACT produced negative results. Sensitivity for stand-alone collimated CACT (CACT_Coll_) could be computed at 0.93, with a total of 74 out of 80 performed CACTs confirming selective cannulation via adrenal vein opacification. Pairwise Wilcoxon signed-rank tests between cortisol concentrations in the right and left adrenal vs. peripheral vein proved significant (*p* < 0.0001).

Weighed against the standard full-FOV CACT in an age- and weight-matched cohort receiving liver-directed therapy at our center, a significant reduction of radiation exposure could be demonstrated with the collimated CACT utilized in this study. While mean DAP_Coll_CACT_ lay at 2414 ± 958 [μGy x m^2^], mean DAP_Full-FOV_CACT_ could be measured at 8766 ± 1956 [μGy × m^2^] (*p* < 0.0001). No significant difference was found in fluoroscopy time, averaging at approximately 23 min. No procedure-related complications such as AV rupture and subsequent intraglandular and widespread periadrenal hemorrhage, adrenal infarction or adrenal vein thrombosis occurred.

## Discussion

In this study, we demonstrate that the acquisition of collimated CACTs in AVS procedures of patients with primary hyperaldosteronism increases the interventional success rates significantly, raising them from an initial 72.1% after sole consideration of a SI_Cortisol_ > 2.0, to 90.1% after combining several hormonal indices with positive imaging data. Furthermore, a significant radiation dose decrease could be achieved by the acquisition of collimated instead of full-FOV CACT in an age- and weight-matched cohort.

Accounting for > 5–10% of hypertensive patients, primary hyperaldosteronism has been recognized as the most frequent form of secondary endocrine hypertension [3, 6, 9, 21, 24]. Higher rates of cardiovascular complications and renal damage compared to essential hypertensives, potentially reversible with surgical or medical treatment, demonstrate the enormous significance of specific and reliable diagnostic tools for differentiating the two most common subtypes, APA and BHA [12, 24]. The high diagnostic accuracy and extremely low rate of complications (0.61%), coupled with an improved outcome of AVS guided surgical management according to a global multicenter study [25] and a large registry study (> 1600) [26], represent crucial assets of AVS and have led to its endorsement in the majority of PA patients, excluding patients unable or unwilling to undergo surgical treatment [3, 13]. Despite this recommendation in the current guidelines by the Endocrine society, it is not universally performed—potentially due a formed impression of AVS as a challenging, complex intervention with a high failure rate, and a tendency towards avoidance of AVS for easier, though less specific alternatives, such as the sole employment of CT/MRI [1, 2, 4, 6, 8–11, 13, 17]. Due to widespread availability and helpful assistance in the surgeon’s understanding of the adrenal vasculature, the aforementioned imaging modalities still play an important role in differentiating unilateral from bilateral forms of PA [1, 2, 4, 6, 8–11, 13, 17]. However, limited specificity has been described for diagnoses exclusively on the basis of CT/MRI, with diverging management recommendations in up to nearly 38% compared to the gold standard (AVS), especially in cases such as microadenomas escaping attention or upon detection of nonfunctioning adenomas [3, 9, 27, 28]. Since misinterpretation of imaging could lead to inappropriate treatment decisions, high diagnostic specificity and reliability are crucial for this potentially curable disease. Different attempts have been made in order to facilitate the catheterization of the adrenal veins, including utilization of quick cortisol assays (QCA) and/or use of advanced CT imaging techniques [7, 8, 12, 18–20]. With particular benefit in centers with initially low success rates according to Betz et al. [18], the QCA method issues an additional need for dedicated equipment and trained personnel within the angiography suite, potentially creating logistical and financial challenges. In our study, we chose to outsource laboratory analysis to the central laboratory unit at our University, resulting in upwards of 60 min of wait time, including transport, centrifugation and measurement. Before the availability of CACT, the patient generally awaited the results of the sample analysis in the radiology suite, thus occupying space and time, in which additional interventions could be performed. This waiting time might be reduced by collimated CACT acquisition, providing feedback concerning catheter positions and alleviating doubts of successful cannulation, especially in less experienced IRs, thus leading to an earlier release of the patient. Other groups have also reported advantages by adding different imaging techniques such as a full-FOV CACT, DSA or fluoroscopy image fusion guidance [7, 8, 11, 15, 17]. In our study of 111 (included) AVS procedures, we could duplicate this effect. However, instead of a full-FOV CACT, we utilized preponderantly right-sided, collimated CACTs in 80 patients with ambiguous hormone indices, thus reducing radiation exposure in both the relatively young patient collective as well as the operator. In two cases, left-sided CACT_Coll_ enabled identification of the correct sampling position, demonstrating a beneficial effect—albeit lower than on the right side. In addition, collimated CACT acquisition significantly improved the success rate of right-sided AVS from an already comparable result of 72.1% at our hospital to slightly over 90%. While this may in part be due to a learning curve, we included a wide variety of interventional radiologists with differing ranges of experience, very likely dispersing the aforementioned habituation effect. The differing levels of experience are especially represented in the undeviating left-sided success rate of 99% compared to a lower right side, which may predominantly be explained with difficulties of right adrenal vein sampling due to highly prevalent anatomical variations (e.g., common-trunk type with accessory hepatic veins vs. independent type), small size and angulations [15]. Depending upon achievement of adequate cortisol gradients between adrenal and peripheral levels, the wide range of success rates reported in literature have likely been impacted by considerable variability in the performance and interpretation of AVS, beginning with fundamental questions such as who should undergo the procedure and how to analyze the results. Further influencing factors that have been described include the type of catheterization, sequential vs. simultaneous stimulation with adrenocorticotropic hormone (ACTH) vs. lack of stimulation, the choice of cutoff values in the lateralization criteria, the circadian variations of cortisol, and patient preparation (dietary restrictions, medication, posture and length of time in that posture) [1, 24]. Though some studies advocate the use of either continuous or bolus cosyntropin administration to minimize stress-induced aldosterone fluctuations and avoid the risk of a falsely negative blood sample during a phase of low aldosterone secretion [15, 17], there is no conclusive evidence of the method’s superiority regarding outcome [5]. It also has been shown that it can result in discordant results pre- to post-stimulation [29] or false lateralisation of PA [24, 30–32]. Therefore, our AVS protocol consisted of an unstimulated sequential AVS which we endeavored to schedule in the morning hours (7–11 a.m.) after a night of recumbency in order to avoid stress-related or expected diurnal fluctuations in ACTH [1, 3]. We employed initial, stand-alone cutoff values of SI_Cortisol, Aldosterone_ ≥ 2 for correct AVS position in this study, as these are the most widely reported values in unstimulated AVS [1, 5, 23, 24, 32]. In addition, Mailhot et al. demonstrated that a combination of both hormone indices with a cutoff value of at least 2 had the best sensitivity for 100% specificity [23]. However, there is no definite agreement on these values, with existing reports of both more restrictive criteria, e.g., SI_Cortisol_ ≥ 3.0 [33] as well as more permissive criteria such as SI _Cortisol_ ≥ 1.1 [34], which can have a significant effect on success rates. Criteria too stringent, meaning a very high cut-off, might result in interpreting an adequate result as failed cannulation, and vice versa. To this end, we chose to adapt the selectivity indices for both, cortisol and aldosterone, to at least > 1.1 after demonstration of an adequate sampling position in the collimated CACT, creating a potential new gold standard for the analysis of AVS results. This was done intending to prevent misinterpretation of values lower than the cutoff due to contralateral suppression. After ensuring a successful cannulation via opacification of adrenal veins, lateralization of excess aldosterone production or unilateral disease was assessed: If the adrenal venous aldosterone/cortisol ratio on one side showed values at least two times greater than the simultaneous peripheral venous ratio (LI ≥ 2) and the adrenal venous ratio on the contralateral side was lower than the peripheral venous ratio (CSI ≤ 1), the study was considered to demonstrate lateralization of aldosterone production. This combination of LI and CSI for the definition of lateralization is based on the concept of renin suppression by unilateral PA leading to a consecutive suppression of aldosterone but not cortisol from the contralateral “unaffected” gland [24]. In addition, computation of the CSI may prove helpful in subtype diagnosis, especially in cases with failed cannulation on one side and a subsequently missing LI [24, 35]. Our failed results in this study might in part be explained by a superselective blood sample from an area not housing the adenoma, venous drainage of the adenoma via veins other than those cannulated or even an ectopic source of aldosterone overproduction [31–33, 36, 37]. This might have been the case in the seven unsuccessful catheterization studies and especially in the four cases receiving a CACT indicating incorrect sampling position in accessory hepatic veins, and consecutive demonstration of negative hormone thresholds. Potential other explanations could be our overall lack of ACTH stimulation and the circadian fluctuations of the hormone concentrations. This might have lowered the overall PCC and PAC gradients between the adrenal vein and the IVC, especially since the majority of the formally negative cortisol indices still lay at approximate values of 2.0 and thus at the precipice of being considered (weakly) positive. While it has been questioned whether sequential catheterization in blood sampling without cosyntropin stimulation might cause artificial gradients due to the pulsatile nature of both cortisol and aldosterone secretion, the usual gap between both cannulations at our center lay between 5 and 10 min due to the experience of the IR and hence might not be significant. In a multicenter study by Mulatero et al. with sequential blood sampling, there was no evidence for significant fluctuations in peripheral aldosterone or cortisol levels when comparing studies at different points in time [15]. In addition, bilateral simultaneous AVS may increase the risk of adrenal vein thrombosis, due to increased time of the catheter’s obstruction of the vessel lumen [5]. Neither adrenal vein thrombosis nor any of the previously published complications, e.g., AV rupture with subsequent intraglandular and widespread periadrenal hemorrhage or adrenal infarction, occurred in our study, leading to an even lower complication rate than the reported 0.61% by Rossi et al. [25], compounding the impression of a procedure with high safety profile. Increased safety also stems from the collimation-based reduction of radiation exposure, with significantly decreased DAP_Coll_CACT_ when weighed against DAP from a suitable control cohort receiving a full-FOV CACT as well as a significant increase of successful cannulation rates to 90.1%. While these are not 100% specific and sporadically hard to differentiate from an accessory hepatic vein or a renal capsular vein [22], venographic features, previously described as typical for the adrenals, were interpreted as successful selective cannulation: opacification of the superficial veins of the adrenal capsule and a gland-like pattern as well as a stellate appearance for the right adrenal, produced by pinnately branching tributaries from the central vein [22]. Novel techniques such as the measurement of steroids (18-oxocortisol, 18-hydroxycortisol) on the basis of mass spectrometry for distinguishing APA from patients with bilateral disease or PET-CT with labelled metomidate has been described, demonstrating that adjuncts and/or alternatives to AVS are being researched to improve the diagnostic process, however, as of yet the findings are still based on limited data and restricted availability [5, 24].

## Limitations

The limitations of this study are based on its retrospective nature and evaluation of a singular procedure in one individual center. A larger population than the included 111 interventions combined with a multi-centric approach, prospective study design and correlation with post-surgical results should be considered, to further assess the potential of collimated CACT on increasing the success rate of selective cannulations in AVS. This did not prove possible in our cohort as the majority of patients are referred to our center solely for the AVS and are operated elsewhere. Furthermore, our results might be influenced by our choice of cutoff-values as well as our execution of the non-standardized AVS procedure by interventional radiologists with varying amounts of experience.

## Conclusion

Collimated CACT of the adrenals is a simple and safe technique that enables intraprocedural confirmation of accurate catheterization, thereby contributing to a significant increase of successful (primarily right-sided) cannulation rates in AVS and offering an alternative to previously published AVS analysis algorithms with lower radiation exposure compared to a full-FOV CACT.

## Abbreviations

ACTH: Adrenocorticotropic hormone
APA: Aldosterone-producing adenoma
AV: Adrenal vein
AVS: Adrenal venous sampling
BAH: Bilateral adrenal hyperplasia
CACT_(Coll, Full-FOV)_: (Collimated, full-FOV) C-arm computed tomography
CSI: Contralateral suppression index
DSA: Digital subtraction angiography
FOV: Field of view
IVC: Inferior vena cava
IR: Interventional radiologist
LI: Lateralization index
PA: Primary hyperaldosteronism
PAC: Plasma aldosterone concentration
PCC: Plasma cortisol concentration
PV: Peripheral vein
QAC: Quick cortisol assay
RAV: Right adrenal vein
SD: Standard deviation
SI: Selectivity index

## References

[1] Blondin D, Quack I, Haase M, Kucukkoylu S, Willenberg HS (2015) Indication and technical aspects of adrenal blood sampling. Rofo 187 (1):19–28. doi:10.1055/s-0034-1385081 10.1055/s-0034-1385081 25226232

[2] Chayovan T, Limumpornpetch P, Hongsakul K (2019) Success rate of adrenal venous sampling and predictors for success: a retrospective study. Pol J Radiol 84:e136-e141. doi:10.5114/pjr.2019.84178 10.5114/pjr.2019.84178 PMC6479143 31019607

[3] Funder JW, Carey RM, Fardella C, Gomez-Sanchez CE, Mantero F, Stowasser M, Young WF, Jr., Montori VM, Endocrine S (2008) Case detection, diagnosis, and treatment of patients with primary aldosteronism: an endocrine society clinical practice guideline. J Clin Endocrinol Metab 93 (9):3266–3281. doi:10.1210/jc.2008-0104 10.1210/jc.2008-0104 18552288

[4] Higashide T, Funabashi N, Tanaka T, Inoue K, Kazama T, Motoori K, Nagano H, Nakatani Y, Ichikawa T, Takaoka H, Uehara M, Yokote K, Kobayashi Y, Uno T (2013) Detection of adrenal veins on selective retrograde CT adrenal venography in comparison with digital subtraction angiography in subjects with established diagnosis of one-sided adrenal aldosterone-producing tumor confirmed by adrenal vein sampling, histopathology and clinical course. Int J Cardiol 168 (4):3254–3258. doi:10.1016/j.ijcard.2013.04.140 10.1016/j.ijcard.2013.04.140 23647597

[5] Rossi GP, Auchus RJ, Brown M, Lenders JW, Naruse M, Plouin PF, Satoh F, Young WF, Jr. (2014) An expert consensus statement on use of adrenal vein sampling for the subtyping of primary aldosteronism. Hypertension 63 (1):151–160. doi:10.1161/HYPERTENSIONAHA.113.02097 10.1161/HYPERTENSIONAHA.113.02097 24218436

[6] Schwab CW, 2nd, Vingan H, Fabrizio MD (2008) Usefulness of adrenal vein sampling in the evaluation of aldosteronism. J Endourol 22 (6):1247–1250. doi:10.1089/end.2008.0007 10.1089/end.2008.0007 18484874

[7] Georgiades C, Kharlip J, Valdeig S, Wacker FK, Hong K (2009) [Use of C-arm CT for improving the hit rate for selective blood sampling from adrenal veins]. Radiologe 49 (9):848–851. doi:10.1007/s00117-009-1865-4 10.1007/s00117-009-1865-4 19697002

[8] Georgiades CS, Hong K, Geschwind JF, Liddell R, Syed L, Kharlip J, Arepally A (2007) Adjunctive use of C-arm CT may eliminate technical failure in adrenal vein sampling. J Vasc Interv Radiol 18 (9):1102–1105. doi:10.1016/j.jvir.2007.06.018 10.1016/j.jvir.2007.06.018 17804771

[9] Kempers MJ, Lenders JW, van Outheusden L, van der Wilt GJ, Schultze Kool LJ, Hermus AR, Deinum J (2009) Systematic review: diagnostic procedures to differentiate unilateral from bilateral adrenal abnormality in primary aldosteronism. Ann Intern Med 151 (5):329–337. doi:10.7326/0003-4819-151-5-200909010-00007 10.7326/0003-4819-151-5-200909010-00007 19721021

[10] Lupi A, Battistel M, Barbiero G, Miotto D, Rossi GP, Quaia E (2019) Simultaneous bilateral adrenal vein sampling for primary aldosteronism: useful tips to make it simple and safe. Eur Radiol 29 (11):6330–6335. doi:10.1007/s00330-019-06209-5 10.1007/s00330-019-06209-5 31025064

[11] Plank C, Wolf F, Langenberger H, Loewe C, Schoder M, Lammer J (2012) Adrenal venous sampling using Dyna-CT--a practical guide. Eur J Radiol 81 (9):2304–2307. doi:10.1016/j.ejrad.2011.05.011 10.1016/j.ejrad.2011.05.011 21620601

[12] Busser WM, Arntz MJ, Jenniskens SF, Deinum J, Hoogeveen YL, de Lange F, Schultze Kool LJ (2015) Image Registration of Cone-Beam Computer Tomography and Preprocedural Computer Tomography Aids in Localization of Adrenal Veins and Decreasing Radiation Dose in Adrenal Vein Sampling. Cardiovasc Intervent Radiol 38 (4):993–997. doi:10.1007/s00270-014-0969-z 10.1007/s00270-014-0969-z 25238715

[13] Funder JW, Carey RM, Mantero F, Murad MH, Reincke M, Shibata H, Stowasser M, Young WF, Jr. (2016) The Management of Primary Aldosteronism: Case Detection, Diagnosis, and Treatment: An Endocrine Society Clinical Practice Guideline. J Clin Endocrinol Metab 101 (5):1889–1916. doi:10.1210/jc.2015-4061 10.1210/jc.2015-4061 26934393

[14] Fallo F, Pilon C, Urbanet R (2012) Primary aldosteronism and metabolic syndrome. Horm Metab Res 44 (3):208–214. doi:10.1055/s-0031-1295412 10.1055/s-0031-1295412 22116746

[15] Mulatero P, Bertello C, Sukor N, Gordon R, Rossato D, Daunt N, Leggett D, Mengozzi G, Veglio F, Stowasser M (2010) Impact of different diagnostic criteria during adrenal vein sampling on reproducibility of subtype diagnosis in patients with primary aldosteronism. Hypertension 55 (3):667–673. doi:10.1161/HYPERTENSIONAHA.109.146613 10.1161/HYPERTENSIONAHA.109.146613 20124107

[16] Omura K, Ota H, Takahashi Y, Matsuura T, Seiji K, Arai Y, Morimoto R, Satoh F, Takase K (2017) Anatomical Variations of the Right Adrenal Vein: Concordance Between Multidetector Computed Tomography and Catheter Venography. Hypertension 69 (3):428–434. doi:10.1161/HYPERTENSIONAHA.116.08375 10.1161/HYPERTENSIONAHA.116.08375 28137990

[17] Morita S, Endo K, Suzaki S, Ishizaki U, Yamazaki H, Nishina Y, Sakai S (2017) Reduction of Radiation Exposure Using Dynamic Trace Digital Angiography and Spot Fluoroscopy During Adrenal Venous Sampling. Cardiovasc Intervent Radiol 40 (5):697–703. doi:10.1007/s00270-017-1567-7 10.1007/s00270-017-1567-7 28138726

[18] Betz MJ, Degenhart C, Fischer E, Pallauf A, Brand V, Linsenmaier U, Beuschlein F, Bidlingmaier M, Reincke M (2011) Adrenal vein sampling using rapid cortisol assays in primary aldosteronism is useful in centers with low success rates. Eur J Endocrinol 165 (2):301–306. doi:10.1530/EJE-11-0287 10.1530/EJE-11-0287 21602315

[19] Mengozzi G, Rossato D, Bertello C, Garrone C, Milan A, Pagni R, Veglio F, Mulatero P (2007) Rapid cortisol assay during adrenal vein sampling in patients with primary aldosteronism. Clin Chem 53 (11):1968–1971. doi:10.1373/clinchem.2007.092080 10.1373/clinchem.2007.092080 17901112

[20] Yoneda T, Karashima S, Kometani M, Usukura M, Demura M, Sanada J, Minami T, Koda W, Gabata T, Matsui O, Idegami K, Takamura Y, Tamiya E, Oe M, Nakai M, Mori S, Terayama N, Matsuda Y, Kamemura K, Fujii S, Seta T, Sawamura T, Okuda R, Takeda Y, Hayashi K, Yamagishi M, Takeda Y (2016) Impact of New Quick Gold Nanoparticle-Based Cortisol Assay During Adrenal Vein Sampling for Primary Aldosteronism. J Clin Endocrinol Metab 101 (6):2554–2561. doi:10.1210/jc.2016-1011 10.1210/jc.2016-1011 27011114

[21] Chang CC, Lee BC, Chang YC, Wu VC, Huang KH, Liu KL, Group TS (2017) Comparison of C-arm computed tomography and on-site quick cortisol assay for adrenal venous sampling: A retrospective study of 178 patients. Eur Radiol 27 (12):5006–5014. doi:10.1007/s00330-017-4930-9 10.1007/s00330-017-4930-9 28677050

[22] Park SI, Rhee Y, Lim JS, Park S, Kang SW, Lee MS, Lee M, Lee SJ, Kim IJ, Lee DY, Cho JS (2014) Right adrenal venography findings correlated with C-arm CT for selection during C-arm CT-assisted adrenal vein sampling in primary aldosteronism. Cardiovasc Intervent Radiol 37 (6):1469–1475. doi:10.1007/s00270-013-0820-y 10.1007/s00270-013-0820-y 24352864

[23] Mailhot JP, Traistaru M, Soulez G, Ladouceur M, Giroux MF, Gilbert P, Zhu PS, Bourdeau I, Oliva VL, Lacroix A, Therasse E (2015) Adrenal Vein Sampling in Primary Aldosteronism: Sensitivity and Specificity of Basal Adrenal Vein to Peripheral Vein Cortisol and Aldosterone Ratios to Confirm Catheterization of the Adrenal Vein. Radiology 277 (3):887–894. doi:10.1148/radiol.2015142413 10.1148/radiol.2015142413 26020437

[24] Wolley M, Thuzar M, Stowasser M (2020) Controversies and advances in adrenal venous sampling in the diagnostic workup of primary aldosteronism. Best Pract Res Clin Endocrinol Metab:101400. doi:10.1016/j.beem.2020.101400 10.1016/j.beem.2020.101400 32115358

[25] Rossi GP, Barisa M, Allolio B, Auchus RJ, Amar L, Cohen D, Degenhart C, Deinum J, Fischer E, Gordon R, Kickuth R, Kline G, Lacroix A, Magill S, Miotto D, Naruse M, Nishikawa T, Omura M, Pimenta E, Plouin PF, Quinkler M, Reincke M, Rossi E, Rump LC, Satoh F, Schultze Kool L, Seccia TM, Stowasser M, Tanabe A, Trerotola S, Vonend O, Widimsky J, Jr., Wu KD, Wu VC, Pessina AC (2012) The Adrenal Vein Sampling International Study (AVIS) for identifying the major subtypes of primary aldosteronism. J Clin Endocrinol Metab 97 (5):1606–1614. doi:10.1210/jc.2011-2830 10.1210/jc.2011-2830 22399502

[26] Rossi GP, Rossitto G, Amar L, Azizi M, Riester A, Reincke M, Degenhart C, Widimsky J, Jr., Naruse M, Deinum J, Schultze Kool L, Kocjan T, Negro A, Rossi E, Kline G, Tanabe A, Satoh F, Christian Rump L, Vonend O, Willenberg HS, Fuller PJ, Yang J, Chee NYN, Magill SB, Shafigullina Z, Quinkler M, Oliveras A, Dun Wu K, Wu VC, Kratka Z, Barbiero G, Battistel M, Chang CC, Vanderriele PE, Pessina AC (2019) Clinical Outcomes of 1625 Patients With Primary Aldosteronism Subtyped With Adrenal Vein Sampling. Hypertension 74 (4):800–808. doi:10.1161/HYPERTENSIONAHA.119.13463 10.1161/HYPERTENSIONAHA.119.13463 31476901

[27] Aono D, Kometani M, Karashima S, Usukura M, Gondo Y, Hashimoto A, Demura M, Furukawa K, Takeda Y, Kawashiri M, Yoneda T (2019) Primary aldosteronism subtype discordance between computed tomography and adrenal venous sampling. Hypertens Res 42 (12):1942–1950. doi:10.1038/s41440-019-0310-y 10.1038/s41440-019-0310-y 31409918

[28] Kline G, Holmes DT (2017) Adrenal venous sampling for primary aldosteronism: laboratory medicine best practice. J Clin Pathol 70 (11):911–916. doi:10.1136/jclinpath-2017-204423 10.1136/jclinpath-2017-204423 28893861

[29] Miyoshi A, Wada N, Baba S, Obara S, Takahashi B, Usubuchi H, Terae S (2020) Left-right differences in adrenal vein sampling for primary aldosteronism. Endocr J 67 (3):327–334. doi:10.1507/endocrj.EJ19-0372 10.1507/endocrj.EJ19-0372 31801916

[30] Seccia TM, Miotto D, De Toni R, Pitter G, Mantero F, Pessina AC, Rossi GP (2009) Adrenocorticotropic hormone stimulation during adrenal vein sampling for identifying surgically curable subtypes of primary aldosteronism: comparison of 3 different protocols. Hypertension 53 (5):761–766. doi:10.1161/HYPERTENSIONAHA.108.128553 10.1161/HYPERTENSIONAHA.108.128553 19349554

[31] Wolley M, Gordon RD, Pimenta E, Daunt N, Slater GJ, Ahmed AH, Stowasser M (2013) Repeating adrenal vein sampling when neither aldosterone/cortisol ratio exceeds peripheral yields a high incidence of aldosterone-producing adenoma. J Hypertens 31 (10):2005–2009. doi:10.1097/HJH.0b013e328362add3 10.1097/HJH.0b013e328362add3 24107732

[32] Wu VC, Yang SY, Lin JW, Cheng BW, Kuo CC, Tsai CT, Chu TS, Huang KH, Wang SM, Lin YH, Chiang CK, Chang HW, Lin CY, Lin LY, Chiu JS, Hu FC, Chueh SC, Ho YL, Liu KL, Lin SL, Yen RF, Wu KD, Group TS (2011) Kidney impairment in primary aldosteronism. Clin Chim Acta 412 (15–16):1319–1325. doi:10.1016/j.cca.2011.02.018 10.1016/j.cca.2011.02.018 21345337

[33] Stowasser M, Gordon RD, Gunasekera TG, Cowley DC, Ward G, Archibald C, Smithers BM (2003) High rate of detection of primary aldosteronism, including surgically treatable forms, after 'non-selective' screening of hypertensive patients. J Hypertens 21 (11):2149–2157. doi:10.1097/00004872-200311000-00025 10.1097/00004872-200311000-00025 14597859

[34] Rossi GP, Sacchetto A, Chiesura-Corona M, De Toni R, Gallina M, Feltrin GP, Pessina AC (2001) Identification of the etiology of primary aldosteronism with adrenal vein sampling in patients with equivocal computed tomography and magnetic resonance findings: results in 104 consecutive cases. J Clin Endocrinol Metab 86 (3):1083–1090. doi:10.1210/jcem.86.3.7287 10.1210/jcem.86.3.7287 11238490

[35] Strajina V, Al-Hilli Z, Andrews JC, Bancos I, Thompson GB, Farley DR, Lyden ML, Dy BM, Young WF, McKenzie TJ (2018) Primary aldosteronism: making sense of partial data sets from failed adrenal venous sampling-suppression of adrenal aldosterone production can be used in clinical decision making. Surgery 163 (4):801–806. doi:10.1016/j.surg.2017.10.012 10.1016/j.surg.2017.10.012 29174432

[36] Daunt N (2005) Adrenal vein sampling: how to make it quick, easy, and successful. Radiographics 25 Suppl 1:S143–158. doi:10.1148/rg.25si055514 10.1148/rg.25si055514 16227488

[37] Kishino M, Yoshimoto T, Nakadate M, Katada Y, Kanda E, Nakaminato S, Saida Y, Ogawa Y, Tateishi U (2017) Optimization of left adrenal vein sampling in primary aldosteronism: Coping with asymmetrical cortisol secretion. Endocr J 64 (3):347–355. doi:10.1507/endocrj.EJ16-0433 10.1507/endocrj.EJ16-0433 28132968

